# The fluence–resolution relationship in holographic and coherent diffractive imaging^1^

**DOI:** 10.1107/S1600576717003065

**Published:** 2017-03-22

**Authors:** Johannes Hagemann, Tim Salditt

**Affiliations:** a Institut für Röntgenphysik, Friedrich-Hund-Platz 1, 37077 Göttingen, Germany

**Keywords:** X-ray near-field imaging, coherent diffractive imaging, dose resolution estimation

## Abstract

This work presents a numerical study of the fluence–resolution behaviour for two coherent lensless X-ray imaging techniques. To this end the fluence–resolution relationship of inline near-field holography and far-field coherent diffractive imaging are compared in numerical experiments. To achieve this, the phase reconstruction is carried out using iterative phase-retrieval algorithms on simulated noisy data. Using the incident photon fluence on the specimen as the control parameter, the achievable resolution for two example phantoms (cell and bitmap) is studied. The results indicate the superior performance of holography compared with coherent diffractive imaging, for the same fluence and phase-reconstruction procedure.

## Abstract 2

In this work the fluence efficiency of two coherent X-ray imaging techniques is studied by numerical experiments. The techniques surveyed are near-field holography and far-field diffraction imaging.

## 1. Introduction   

The simple question ‘What resolution do I get for the invested photon fluence?’ is extremely important for X-ray imaging of radiation-sensitive specimens, such as biological cells and tissues. Structure analysis by diffraction is in general based on elastic scattering of photons and hence on the Thompson scattering cross section, which is much smaller than the cross section for photon absorption. This results in significant energy uptake within the sample and hence causes radiation damage. However, for coherent imaging the dose issue is accentuated, since the information is collected from a single copy of the imaged structure, rather than from a large ensemble of identical constituents over which the dose is distributed. Pioneering studies have addressed this topic within the framework of kinematic scattering theory for far-field coherent diffractive imaging (CDI) (Shen *et al.*, 2004 ▸; Howells *et al.*, 2009 ▸) and have found a steep power law, 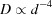, relating dose *D* and resolution *d* for the case of imaging three-dimensional structures at isotropic resolution. Note that this corresponds to equivalent imaging of two-dimensional slices of a width which is scaled down with *d* and hence loses contrast. Conversely, for constant width, increasing only the two-dimensional resolution yields 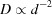 for diffraction as for absorption (see *e.g.* Kirz *et al.*, 1978 ▸). Further work has studied the effect of having a certain feature of interest embedded in other structures (matrix) (Schropp & Schroer, 2010 ▸), showing that the reconstruction quality in CDI is nearly independent of the surroundings (for a given dose). Before addressing the case of (coherent) diffraction, which became important after the advent of CDI (Miao *et al.*, 1999 ▸), earlier work had already compared X-rays, neutrons and electrons as microscopy probes, but had exclusively considered image formation by absorption (Henderson, 1995 ▸). This is understandable for the simple reason that X-ray microscopy started in absorption contrast, and was only later extended to (phase contrast) diffractive imaging. A comparison between X-ray microscopy in absorption contrast (water window spectral range) and by numerical simulation was provided by Huang *et al.* (2009 ▸), showing that isolated low-*Z* materials such as biological cells can be imaged with fewer photons by CDI.

The literature cited above already illustrates the large range of perspectives which one can take to address the dose and resolution issues, at least in a broad sense. One can compare different probes (X-rays *versus* other probes), different types of contrast (absorption *versus* phase contrast), different experimental parameters (notably wavelength) or different types of imaging (*e.g.* lens-based X-ray microscopy *versus* lensless diffractive imaging). To this list, we here add the optical regime of a coherent diffractive imaging experiment.

**Figure 1:**
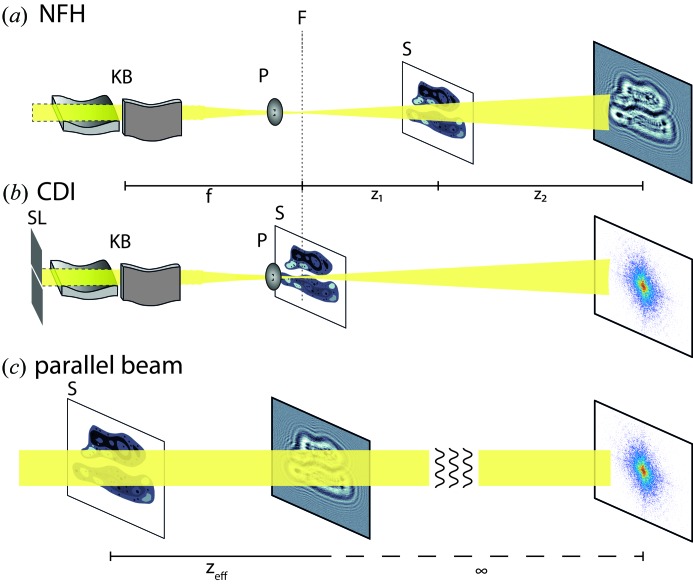
Experimental implementations for (*a*) NFH and (*b*) CDI, shown in a diverging beam geometry (in particular for a synchrotron setup; *cf.* Salditt *et al.*, 2015 ▸). The beam is focused by Kirkpatrick–Baez mirrors (KB), then a pinhole (P) is commonly used as a low-pass filter for removing high spatial frequencies in the probing beam. For CDI the specimen (S) is placed in the focal plane (F), while for NFH it is placed at a defocus position *z*_1_. The detector is then placed at a distance *z*_2_ behind S. This yields for NFH a (de-)magnification of *M* = (*z*_1_ + *z*_2_)/*z*_1_. Note that in order to satisfy the coherence requirements for CDI the effective source size has to be reduced by slits (SL). (*c*) The imaging configurations transferred to the setting of a collimated (parallel) beam. This is achieved *via* a simple coordinate transform (*i.e.* Fresnel scaling; Paganin, 2006 ▸), where the effective propagation distance is given by *z*_eff_ = *z*_2_/*M*.

Notably, we want to compare direct reconstruction of lens­less coherent imaging data in the near-field and far-field regimes. While the previous studies addressing CDI mentioned above were concerned with far-field diffraction, the numerical simulations used in this work are carried out in the optical near-field regime. Fig. 1 ▸ shows a sketch for (*a*) a near-field inline holographic (NFH) imaging experiment and (*b*) a CDI experiment. Fig. 1 ▸(*c*) shows both cases tranferred to the parallel-beam setting, as used in the numerical experiments of this study. Our goal is to provide a quantitative comparison between NFH (Kellström, 1932 ▸; Gabor, 1948 ▸; Howells *et al.*, 1986 ▸; Snigirev *et al.*, 1995 ▸) and CDI (Miao *et al.*, 1999 ▸; Robinson & Miao, 2004 ▸). The main difference between this work and the above-mentioned studies is thus the imaging regime in use.

Further, a recent numerical study (Villanueva-Perez *et al.*, 2016 ▸) also assessed NFH and CDI and formulated a signal-to-noise criterion. Rather than resolution as in the present study, Villanueva-Perez and co-workers focused on the sensitivity with respect to the phase shift of a given feature and its size at a constant fluence.

The motivations for this study are the experimental indications for the high dose effectiveness of NFH imaging (Bartels *et al.*, 2015 ▸; Wilke *et al.*, 2014 ▸; Jones *et al.*, 2014 ▸). In the work of Bartels *et al.* (2015 ▸) for example, NFH images of bacteria were recorded in the multi-keV regime, where a single bacterium is essentially a pure phase-contrast object. Reconstructions were obtained at a dose which was orders of magnitude smaller than reconstructions of similar resolution obtained previously for the same bacteria by (far-field) ptychography (Wilke *et al.*, 2012 ▸). Since experimental work can always be influenced by a number of additional parameters which can, for example, easily render the data in­consistent, a higher or lower dose required for a particular experiment is not conclusive *per se*. In the light of the limited evidence, we therefore turn to numerical analysis, comparing CDI with NFH for simulated noisy data on the same phantoms. To this end, we first used the maximum-likelihood (ML) approach introduced by Elser & Eisebitt (2011 ▸). Accordingly, a critical fluence μ_c_ can be defined, above which the correct phantom (random bitmap) out of a selection of random bitmaps could be identified with a chosen tolerance (error) level ∊ and for given photon shot noise (Jahn *et al.*, 2017 ▸). In this way, one can test the information content in noisy two-dimensional diffraction patterns and investigate the dependence of μ_c_ on object contrast levels, the accepted error level and the bitmap size. For the experimentally relevant case of weak phase contrast, applicable to most biological samples, NFH required a lower dose than CDI for the optimum propagation distance (Fresnel number) (Jahn *et al.*, 2017 ▸). However, apart from small oscillations in μ_c_ as a function of distance, as expected based on the contrast transfer function, the results in the near field were almost identical to the far-field results (Jahn *et al.*, 2017 ▸). Hence, as far as the encoding of information is concerned, which can be tested by the ML approach, far-field CDI and near-field NFH seem in principle to be roughly equal in dose efficiency. What the ML approach cannot address, however, is whether an unknown object can actually be reconstructed from the noisy data, rather than just comparing likelihoods between the true object and some alternatives (bitmaps with randomly switched bits).

**Figure 2:**
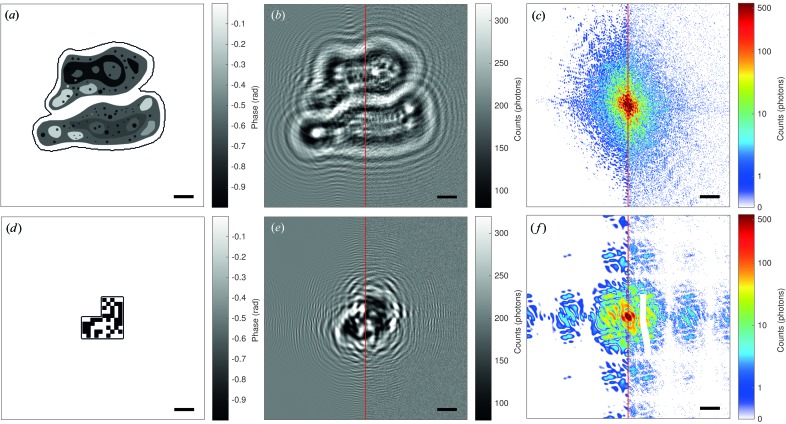
The setup of the numerical experiment. (*a*) The phase-only phantom of two cells with individual compartments and maximum (exaggerated) phase shift of −1 rad. The size of the phantom is 512 × 512 pixels embedded in 1024 × 1024 pixels. (*b*) Simulated near-field intensity measurement at a Fresnel number of 10^−3^ (linear scaling). The left half shows the noiseless measurement, while the right-hand side shows the measurement with noise for 200 photons per pixel in the detection plane. (*c*) Simulated far-field intensity measurement, analogous to panel (*b*); a count of zero photons corresponds to white (logarithmic scale). (*d*) Bitmap pattern analogous to Jahn *et al.* (2017 ▸). The maximum phase shift is −1 rad and the size of one bitmap pixel is represented by 10 × 10 pixels in the sample plane. (*e*), (*f*) Analogous to panels (*b*) and (*c*), respectively. The solid line surrounding the objects in panels (*a*) and (*d*) marks the border of the support used in the reconstruction. The scale bar indicates 50 pixels.

In this work, we fill this gap and actually test the real process of reconstruction from noisy diffraction patterns and not just an ML reconstructability criterion. The main control parameter in this numerical work is again the fluence μ, *i.e.* the average number of photons per pixel in the plane of the object. Using μ we are able to tune our numerical experiment from the case ‘barely reconstructing’ to ‘best object reconstruction’. According to this parameter we generate test data of two phantoms: (i) a cell, and (ii) a bitmap object [as done by Jahn *et al.* (2017 ▸)] (see Fig. 2). Following the generation of these noisy diffraction patterns, we run phase-retrieval algorithms on the data and determine the resolution by Fourier ring correlation (FRC) (Harauz & van Heel, 1986 ▸; van Heel & Schatz, 2005 ▸). Appendix *A* [App appa] presents a benchmark of the NFH/CDI propagator without the need of a phase reconstruction. §2 gives details of the data generation and reconstruction scheme. §3 presents the results of the comparison of NFH and CDI. The paper closes in §4 with a summary and outlook.

Of course, implementing both NFH and CDI on the same sample can be experimentally challenging owing to limitations of the setup (coherence, beam size, sampling constraints). These considerations are beyond the scope of this work. Moreover, we consider only coherent scattering (elastic Thomson scattering) and no further interactions of the radiation with matter. Our main focus is the optical regime and the decoding of (phase) information. Thus all simulations are carried out in a dimensionless setting (pixel units and Fresnel number), as detailed below.

## 2. Numerical setup   

Fig. 2 ▸ introduces the concept of the numerical study. In essence the two optical setups – far-field CDI and near-field holography (NFH) – are simulated for two different phantoms, namely a phantom of two adhering biological cells [as used by Giewekemeyer *et al.* (2011 ▸)] and a random binary bitmap; see Figs. 2 ▸(*a*) and 2 ▸(*d*), respectively. Both phantoms are pure phase-contrast objects, with phases 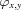 in the range [−1, 0] rad (cell) and 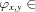 {0, −1} rad (bitmap). Note that binary bitmaps with no correlations between pixels are to some extent amenable to analytical treatments and have been used before, for example by Elser & Eisebitt (2011 ▸) (one-dimensional bitmap) and by Jahn *et al.* (2017 ▸) (two-dimensional bitmap).

Both images have a size of 512 × 512 pixels embedded in 1024 × 1024 (*N_x_* × *N_y_*) pixels. This embedding ensures that the simulated numerical aperture (NA) is sufficently large to recover details down to the pixel level. The NA in a vacuum is given by the opening angle α of the detector,
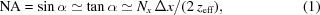
where paraxiality is assumed and 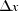 is the pixel size. The resolution limit 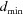 due to the NA is 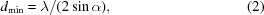
with wavelength λ. Inserting equation (1) and using the definition of the Fresnel number Fr = 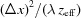 yields 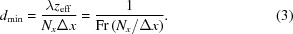
Setting 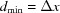 thus yields Fr 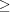 1/*N* as a requirement for the NA.

In both CDI and NFH, we assume perfect illumination by a point source or in equivalent geometry by a plane wave (*cf*. Fig. 1 ▸ *c*), such that the exit wave Ψ is given by the phantom Ψ = 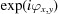. The measurements for NFH were generated by applying the Fresnel propagator 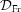 given by 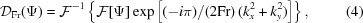
where 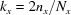 and 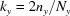 are spatial frequencies in Fourier space with *n*_*x*,*y*_ 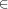 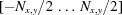 and a Fresnel number of Fr = 10^−3^. The measurements for CDI were generated by discrete Fourier transformation 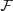 of the corresponding exit wave. Next, the generated intensity patterns (far-field and near-field, respectively) in the detection plane were subjected to Poissonian noise using the routine imnoise (Matlab Inc.), with the average photon fluence μ (in photons per pixel) in the object plane as the only parameter. Figs. 2 ▸(*b*) and 2 ▸(*c*), and 2 ▸(*e*) and 2 ▸(*f*), show in each case the ideal noiseless simulated data or ‘measurements’ (left-hand side) and a random realization of the noisy measurement for a fluence of 200 photons per pixel (in the exit plane; right-hand side). The example NFH and CDI measurements simulated for the phantom nicely illustrate the completely different nature of the signals. In the case of NFH, the signal varies around one (normalized primary beam) by self-interference of the primary beam with the diffracted beam behind the object, and is best represented on a linear scale with a narrow range of intensities. In contrast, the CDI data cover many orders of magnitude from the central pixel to the edge of the detector, where most pixels have zero photon counts. Note that in this idealized simulation we take the full diffraction pattern into account, *i.e.* the numerical aperture is sufficiently large, and we assume that the detector does not need any kind of beamstop, which would result in a loss of information.

Thus, in summary, the noisy measurements were generated using the following recipe:

(i) Propagate the field Ψ from the sample plane to the measurement plane (detection plane) using the respective propagator 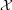 (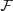 or 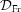).

(ii) Calculate the intensities of the field, yielding the measurement *M* = 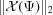. Normalize *M* so that *M*′ = 
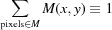
.

(iii) Multiply *M*′ by μ*N_x_N_y_* and use the result as input for a Poisson random number generator. This yields the noisy measurement used in the phase reconstruction.

The reconstructions from the noisy data were obtained using the relaxed averaged alternating reflections (RAAR) algorithm (Luke, 2005 ▸). The iterations are given by 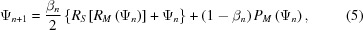
where 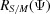 = 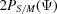 − Ψ denotes a (mirror) reflection by a given constraint set and *n* the iteration index. The parameter β_*n*_ controls the relaxation. It follows the function 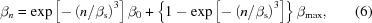
where β_0_ denotes the starting value, β_max_ the final value of β_*n*_ and β_s_ the iteration number when the relaxation is switched. This relaxation strategy follows equation (37) of Luke (2005 ▸). The parameters were set for β_0_ = 0.99, β_max_ = 0.75 and β_s_ = 150 iterations for all reconstructions. The projection on the measurements 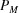 is the standard magnitude projector
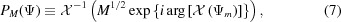
where 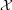 is either 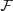 or 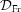 for far-field or near-field propagation, respectively. In equation (74) of Luke *et al.* (2002 ▸) an alternative version of 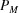 is given which should handle numerical inconsistencies such as noise. In our case, experiments using this version did not show any improvement in the resolution.

The operator 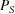 is used to enforce the support *S*, which is assumed to be perfectly known, and the pure phase constraint in the object plane, *i.e.*

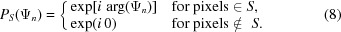
Details on the implementation can be found in the supporting information.

## 3. Results   

**Figure 3:**
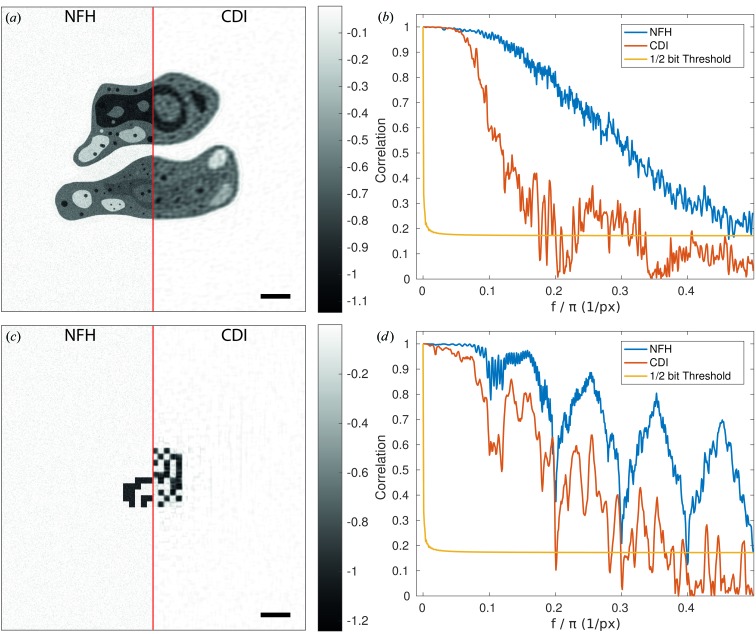
Example reconstructions for 200 photons per pixel. (*a*) The phases (radians) of the reconstruction of the cells after 200 iterations of RAAR (β_0_ = 0.99, β_max_ = 0.75 and β_s_ = 150 iterations) using a support and pure phase object constraint. The left half shows the reconstruction obtained from the near-field data, the right-hand side the results for the far-field data. (*b*) Fourier ring correlation of the reconstructions with the phantom in Fig. 2 ▸(*a*). (*c*), (*d*) Analogous to panels (*a*) and (*b*) for the bitmap object shown in Fig. 2 ▸(*c*). The scale bar indicates 50 pixels.

Before addressing the fluence–resolution relationship, we present an example reconstruction to illustrate the steps that are necessary to obtain the reconstruction data, which are then analysed by massively parallel batch processing. Fig. 3 ▸ shows an example reconstruction for μ = 200 photons per pixel for both phantoms (Figs. 3 ▸ *a* and 3 ▸ *c*), using the noisy measurements shown in Figs. 2 ▸(*b*), 2 ▸(*c*), 2 ▸(*e*) and 2 ▸(*f*).

The left- and right-hand sides of Figs. 3 ▸(*a*) and 3 ▸(*c*) show the phases of the NFH and CDI reconstructions, respectively, again for both (*a*) the cell and (*c*) the bitmap phantom. The reconstructions are based on the same set of parameters and constraints as far as possible. There are two differences: (i) the propagation operator (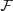 or 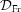) and (ii) the starting guess. For the holographic reconstruction, an array uniformly initialized with amplitude 1 and phase 0 was used, whereas the CDI reconstruction used an initialization consisting of uniform amplitude 1 but randomly chosen phases from the range 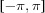, both in the object plane. Changing the initial guess typically had only a small effect on the results. Inspection of the holographic reconstructions in Figs. 3 ▸(*a*) and 3 ▸(*c*) shows some high-frequency noise in the background of the reconstructions, but the fine structures of the cells (small black dots) are still clearly visible and the edges of the bitmap are still sharp. The CDI reconstruction of the cell clearly shows a loss of detail, but the background is less noisy. For the bitmap we see washed out edges and some structured background which matches the length scale of the bits. Note that the reconstructions show the object after the measurement projection (before the support is enforced).

For a quantitative comparison of the resolution we used FRC. To this end, the phases of the reconstructions were correlated with the corresponding phantom phases (Fig. 2 ▸), and the decrease in correlation was plotted as a function of spatial frequency. The resolution is determined by the first intersection of *f*_r_, the resolved spatial frequency, with the so-called half-bit threshold, indicating the degree of correlation at which sufficient signal has been acquired (van Heel & Schatz, 2005 ▸). The results are shown in Fig. 3 ▸ for (*b*) the cell and (*d*) the bitmap. The FRC curve in Fig. 3 ▸(*b*) for the CDI reconstruction decays much faster (*f*_r_ = 0.17) than for the NFH reconstruction (*f*_r_ = 0.48), in agreement with visual inspection. Interestingly, the FRC curves for the bitmap phantom show an oscillatory behaviour, but again the CDI curve decays faster (*f*_r_ = 0.2) than the NFH curve (*f*_r_ = 0.4), *i.e.* it shows lower resolution.

**Figure 4:**
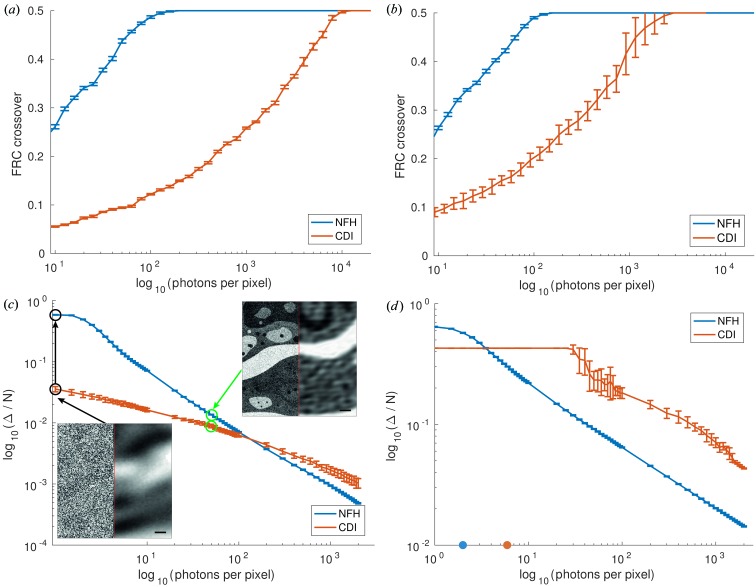
Resolution as a function of dose for holographic and coherent diffractive imaging. (*a*) The result for the cell phantom. (*b*) The result for the bitmap phantom. The photon number ranges from 1 to 20 000 photons per pixel. Each photon number had 30 realizations for the near- and far-field measurements. The reconstructions were carried out with RAAR and the same settings as before. (*c*) Δ (normalized by the number of pixels in the support *N*) for the cell phantom as a function of fluence. The insets (200 × 200 central pixels of the cell phantom) show different phase reconstruction snapshots (NFH left, CDI right) for fluences of 1 (black) and 50 (green) photons per pixel, with respectively coloured arrows. The inset scale bar is 20 pixels. (*d*) Δ/*N* for the bitmap. The coloured dots mark the critical fluence obtained by ML simulations.

Next, we turn to the fluence–resolution relationships, which were computed by performing the automated reconstruction and FRC analysis for measurements of systematically varied fluence μ. For each μ covering the range from 1 to 20 000 photons per pixel (phantom plane), 30 realizations were generated and reconstructed, each with the same parameters. Fig. 4 ▸ shows the results. Comparing the results for (*a*) the cell and (*b*) the bitmap, we notice that in both cases the NFH reconstruction reaches the maximum achievable resolution at a significantly smaller fluence. Note that the spatial frequency of 0.5 periods per pixel corresponds to the maximum (half-period) resolution of a pixel. However, reaching the full resolution does not necessarily equate to having a perfect reconstruction. For example, the l2-norm of the difference image (reconstruction phantom) can be non-zero, while the FRC has already saturated. Comparing Figs. 4 ▸(*a*) and 4 ▸(*b*), we notice that NFH reaches a maximum resolution for both objects at the same fluence of around 300 photons per pixel. At the same time, the error bars of the bitmap results are larger than those for the cell. In contrast, CDI needs 11 000 photons to reach full resolution for the cell, and 3000 photons for the bitmap. Furthermore, we analysed the error of the reconstruction by the l2-norm [see Fig. 4 ▸(*c*) for the cell and Fig. 4 ▸(*d*) for the bitmap]. To this end, the l2-norm Δ of the phase difference, 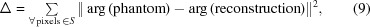
was computed for all pixels within the support. The Δ curves in Figs. 4 ▸(*c*) and 4 ▸(*d*) are normalized by the number of pixels in *S* and show an unexpected behaviour. At low fluences the error in CDI is smaller than that in NFH, but there is a crossover at μ = 140 (cell) and μ = 4 (bitmap), where NFH becomes superior in terms of Δ. On closer inspection of the reconstruction result, however, it becomes clear that the smaller Δ at low fluence is misleading. CDI yields an unstructured reconstruction with no representation of structural details [see insets in Fig. 4 ▸(*c*)]. The reconstructions are much worse than the NFH results for the same fluence, but exhibit a smaller Δ by way of averaging the signal deviations. We must conclude that Δ is not a well suited error metric at low fluence.

**Figure 5:**
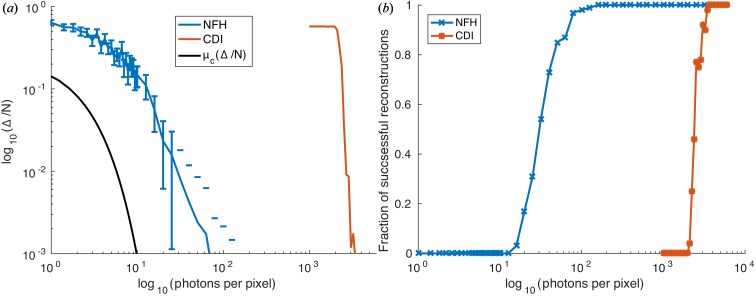
Reconstructability results for the bitmap. (*a*) Δ/*N* evolution for the thresholded reconstruction on a bitmap object with 1:1 pixel correspondence of bitmap and object plane pixels. (*b*) Fraction of successful, *i.e.* Δ = 0, reconstructions for 100 repetitions of a given fluence (*cf*. Jahn *et al.*, 2017 ▸).

Thus, it becomes clear that in all cases tested, NFH yields superior results to CDI. Note that the absolute Δ values also depend on the number of iterations (200 in both cases). Running the algorithm for more iterations, *e.g.* 800, led to further reductions in Δ of about 30% in the case of NFH and 10% for CDI (cell phantom). Furthermore, the introduction of additional constraints can of course also change the error value. For example, using the prior knowledge that the binary bitmap must have discrete phase values 0, −1 suggests the use of a thresholding constraint (binary value projector) 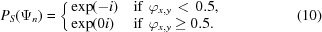
Fig. 5 ▸ shows the results using this projection in addition to the support and pure phase constraint for the bitmap phantom. For these results we used a bitmap with 1:1 pixel correspondence of bitmap to object plane pixel. Thus the entire object has a size of only 10 × 10 pixels. Fig. 5 ▸(*a*) shows the Δ/*N* error [as in Fig. 4 ▸(*d*), but now after the threshold constraint], corresponding to the fraction of wrong pixels. Here we see the expected behaviour in that NFH reconstructs better at low fluence than CDI. For comparison, we also plot the theoretical function μ_c_(Δ/*N*) (solid line) based on the ML analysis, describing the critical fluence to identify the correct bitmap from the noisy diffraction pattern (CDI) out of a set of neighbouring bitmaps (Jahn *et al.*, 2017 ▸). Fig. 5 ▸(*b*) shows the fraction of successful reconstructions, *i.e.* the fraction of successful reconstructions (Δ = 0) from an ensemble of 100 runs as a function of fluence. Comparing these results, we see that CDI reconstructions require substantially more flux at any error level. Furthermore, the functional form of the curve is smoother for NFH, while the transition from non-reconstructible to reconstructible is extremely sharp for CDI, similar to a phase transition.

## 4. Summary and outlook   

In this work, we have investigated the fluence efficiency of variants of lensless X-ray imaging techniques, notably coherent diffractive imaging operating in the optical far field, and inline holography operating in the optical near field. Despite the entirely different nature of the signals and the modes of image formation, which can be classified as heterodyne and homodyne, *i.e.* with and without adding a reference wave, it is commonly assumed that the information contents in the diffraction pattern for a given photon fluence should be equal. The analytical work and simulations of Jahn *et al.* (2017 ▸) have already pointed out that this can never be exactly true, since the oscillatory nature of the contrast transfer functions in NFH results in a dependence on the Fresnel number. Therefore, absorption and phase contrast have to be distinguished, and also the regime of weak or strong contrast. However, the maximum-likelihood approach of Jahn *et al.* (2017 ▸) addresses the information content of the noisy pattern and not the reconstruction quality, which can actually be obtained by standard methods of iterative algorithms. As we have shown here, the latter case is characterized by substantial differences between NFH and CDI. In other words, while the information content may be similar, the ability of the algorithms to decode the diffraction pattern deviates significantly. These conclusions have been substantiated both by the error metric of the l2-norm Δ and by Fourier ring correlation. For example, Fig. 5 ▸(*b*) shows that both NFH and CDI reach full reconstructability within one decade of photon fluence, but for CDI the fluence curve was shifted up by two decades. Furthermore, both NFH and CDI reconstructions required substantially higher fluence, as predicted by the ML approach. In conclusion, our findings point to an important advantage of NFH, in addition to its large tolerance for partial coherence, its compatibility with extended specimens and its flexibility in reconstruction constraints, *e.g.* the pure phase constraint is often sufficient to reconstruct at least a coarse image of the object. Some of these advantages may also apply to ptychography, for example when mixed states are taken into account (Thibault & Menzel, 2013 ▸). It goes without saying that these conclusions await further validation by other reconstruction codes, as well as by careful experimental testing. If the evidence is substantiated, more imaging experiments of radiation-sensitive specimens such as biological objects should be carried out in the holographic regime, for which dedicated synchrotron beamlines are now available.

## Supplementary Material

Simulation details and implementation. DOI: 10.1107/S1600576717003065/vc5008sup1.pdf

Scripts. DOI: 10.1107/S1600576717003065/vc5008sup2.zip

## Figures and Tables

**Figure 6:**
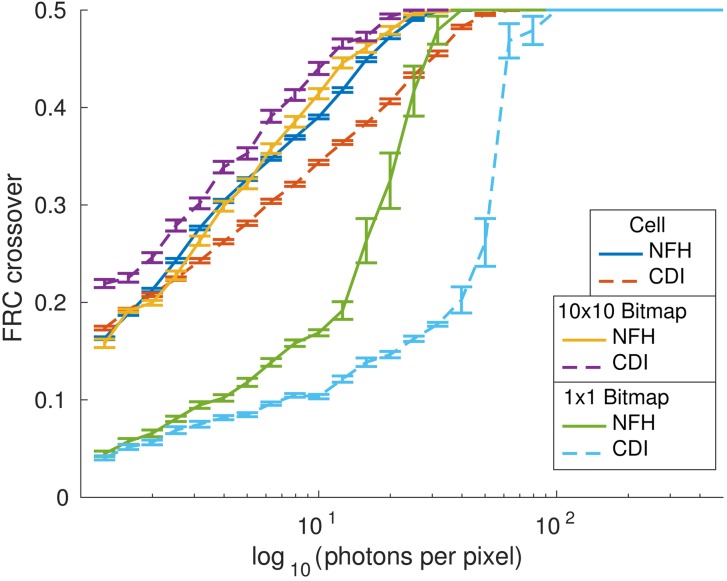
Results for direct back propagation. Three objects are compared: the cell, a bitmap with each bitmap pixel represented by 10 × 10 image pixels, and a bitmap with direct 1:1 pixel correspondence. Solid lines correspond to NFH results for a Fresnel number of 10^−3^ and dashed lines correspond to CDI results. The results for each fluence μ have been averaged over 30 noise realizations.

## Appendix A Direct back propagation

Following the suggestion of a reviewer, we have investigated to what extent the results obtained in this work depend on the reconstruction (which always requires a specific choice of constraints and reconstruction algorithm) and we have also performed simulations in the simplest possible setting, based on direct back propagation. To this end, the exit wave in the sample plane was first propagated to the detector plane to generate diffraction data as before. Then Poisson noise was added to the intensity (amplitude), again as before. Finally, the wave with noisy amplitude and ideal phase was propagated back to the sample plane. The idea of this procedure was to eliminate the role of the reconstruction algorithm used. The ideal complex-valued data *M* is given as

where  is either  or  for NFH or CDI, respectively, and Ψ is the exit wave in the sample plane. Using the noising procedure from the main text gives the noisy intensities , which are then combined with the phases to yield ,

*i.e.* the noisy complex amplitudes. The  are generated for different fluences μ ranging from 1 to 1000 photons per pixel and are then used as input for the inverse propagator  for NFH and CDI. Fig. 6 ▸ shows the results of this numerical experiment. We compare three different phantoms: (i) the cell phantom, (ii) the bitmap phantom with one bitmap pixel represented by 10 × 10 image pixels (oversampled information) and (iii) one bitmap pixel represented by one image pixel.

NFH again shows superior performance for the cell phantom and the 1 × 1 bitmap, but not to the same degree as before. Furthermore, CDI reaches a higher resolution at a lower dose for the 10 × 10 bitmap. This can be understood based on the fact that in this numerical experiment the phases are given, and the main advantage of NFH in encoding phase information by interference does not play a role.

In addition, we have varied the contrast of the object. The results illustrate the same trend for both CDI and NFH: as the phase shift of the phantom increases, fewer photons are needed to reach maximum resolution, as expected. This is in agreement with the findings of Villanueva-Perez *et al.* (2016 ▸).
