# Supplementary material for: The fluence–resolution relationship in holographic and coherent diffractive imaging
Source: J Appl Crystallogr. 2017 Mar 22;50(Pt 2):531–8. doi: 10.1107/S1600576717003065 (PMC5377347; doi:10.1107/S1600576717003065)
Supplement: Supplementary file 1 [file j-50-00531-sup1.pdf]

The Fluence-Resolution Relationship in Holographic and Coherent Diffractive Imaging: Supplement Information

Johannes Hagemann, Tim Salditt (tsaldit@gwdg.de)
Universität Göttingen
Institut für Röntgenphysik,
Friedrich-Hund-Platz 1
37077 Göttingen Germany

March 14, 2017

1 Simulation Details and Implementation

Here we want to give a short overview of the numerical implementation of the simulation using MATLAB (2016a). When the numerical experiments have been set up different phase retrieval algorithms have been tested as alternating projections (e.g. Gerchberg-Saxton and error-reduction algorithm) and the hybrid-input-output algorithm. The results have shown that for our setup, the number of iterations and constraints in use RAAR yielded the best results. Listing 1 shows the minimal implementation of RAAR, which lacks for example the error control. The simulations are sped-up by the use of GPUs. In order to use GPUs more efficiently some data is moved explicitly in the RAM of the GPU e.g. lines 9 and 10. Listing 2 shows the near-filed implementation of RAAR. Only the main differences are shown, these are (i) an additional call parameter `F` (ii) the modified P_M for which we use 2 `PropagatorGPU` objects, for forward and backward propagation.

Lsting 3 shows the wrapper function which generates the test data and carries out resolution analysis. The structure of this script is the following first we set some parameters L. 6-20, then the phantom is chosen and set up L. 21-44. Then the NFH reconstruction and FRC calculation is carried out L. 46-67 followed by the CDI reconstruction L. 70-93. We note that CDI reconstructs the object somewhere in the support thus it is necessary to align the reconstruction with the phantom in order to get correct resolution and error measurements. NFH is in this aspect much more indulgent. The code and helper functions can be found on GitHub: `https://github.com/JHoahg/Resolution-and-Fluence`.

Listing 1: Implementation of RAAR for the far field (`RAAR_ff.m`).

```
function [result] = RAAR_ff(M, psi, iterations, p)
% inputs:
% M              - measurement (amplitudes)
% psi            - initial guess
% iterations     - number of iterations
% p              - parameters

h = waitbar(0, 'progress');

% get input arrays and put them on GPU
M = gpuArray(M);
supp = gpuArray(logical(p.supp));

% Amp_valid can be used to discriminate bad pixels
Amp_valid = gpuArray(p.Amp_valid);
psi = gpuArray(psi);

%  algorithm parameters from data struct p
b_0 = p.b_0;
b_m = p.b_m;
b_s = p.b_s;

for ii = 1:iterations
    waitbar(ii / iterations, h, ...
        sprintf('%d / %d',ii, iterations));

    % relaxation parameter for current iteration cf. Eq. (??)
    b = exp(-(ii/b_s)^3)*b_0 + (1 - exp(-(ii/b_s)^3))*b_m;

    psi_old = psi;

    % P_M
    psi = DFT(psi);
    psi(Amp_valid) = psi(Amp_valid)./ abs(psi(Amp_valid)) ...
             .* M(Amp_valid);
    P_M = IDFT(psi);

  % R_M
    R_M = 2* P_M - psi_old;

    % P_S
    % pure phase constraint
    psi(supp) = exp(1i .* angle(R_M(supp)));
    % support and negativity constraint
    psi(~supp | angle(psi) > 0) = 1;

  %  R_S
    psi = 2*psi - R_M;

    %  new RAAR iterate cf. Eq. (??)
    %  psi = R_S(R_M(Ψ_n)) , psi_old = Ψ_n
    psi = (b/2) * (psi + psi_old) + (1-b)*P_M;

end
%final projection on M

result = gather(psi);
close(h);
end
```

Listing 2: RAAR near field implementation, only differences are shown (`RAAR_nf.m`).

```
function [result] = RAAR_nf(M, psi, iterations, F, p)
% ... Same as in RAAR_ff.

% Propagator objects
prop = PropagatorGPU(F, F, size(M,2), size(M,1));
prop_back = PropagatorGPU(-F, -F, size(M,2), size(M,1));

% ...
for ii = 1:iterations
% ...
    % P_M
    psi = prop.propTF(psi);
    psi(Amp_valid) = psi(Amp_valid)./ abs(psi(Amp_valid)) .* M(
    Amp_valid);
    P_M = prop_back.propTF(psi);
% ...
end

end
```

Listing 3: Usage example of data generation, algorithms and analysis (`fluence_cdi_vs_nfh.m`).

```

% Setup directories, paths, standard settings...

%% Simulation parameters
% sizes of image and surrounding frame
p.width = 512;
p.height = 512;
p.width2 = 1024;
p.height2 = 1024;
p.rec_width = 1024;
p.rec_height = 1024;

p.F = 1e-3;
p.num_photons = 200;
p.b_0 = 0.99;
p.b_m = 0.75;
p.b_s = 150;
p.num_iterations = 200;
p.Amp_valid = logical(true(p.rec_height, p.rec_width));

%% Sample Setup - choose dicty or bitmap
if(1) %dicty sketch
  %prepare probe reads the images and scales gray values to desired
     phase/amplitude values.
    sample = prepare_probe('dicty_sketch.png', 'dicty_sketch.png',
     0, 1, 1, 1, p);
    sample = abs(sample) .* exp(1i.*(angle(conj(sample))));
    p.supp = load('support_dicty_ff.mat');
    p.supp = p.supp.supp.I_supp;
    p.supp = imresize(p.supp, (p.height/256), 'box');
end
```

```
if(0) % use binary bitmap
    upscale =  10;
    sample = binary_bitmap(10, 10, -1, upscale, p.rec_height, p.rec_width);
    p.supp = ones(10*upscale, 10*upscale);
    p.supp(1:floor(10*upscale/2), 1:ceil(10*upscale/2)) = 0;
end

p.supp = pad_to_size(p.supp, p.rec_height, p.rec_width, 'zero');

figure()
imagesc(angle(mid(sample,p)));

figure()
imagesc(mid(p.supp, p));

%% NFH data setup, as described in the recipe in Sec. 2
prop = Propagator(p.F, p.F, p.width2, p.height2,1);
id_holo = abs(prop.propTF(sample)).^2;
id_holo = id_holo ./ sum(id_holo(:));
figure; imagesc(id_holo)

if(1) % use RAAR
  holo = imnoise((id_holo*p.num_photons.* numel(id_holo))*1e-12, 'poisson')*1e12;
  % set 0 to epsilon, otherwise we get NaNs
  holo(holo == 0) = 2*eps;
  % this rescaling is neccessary to get float numbers again, the reconstruction did not work (also for CDI) for integer numbers in the measurement.
  holo = holo ./ sum(holo(:)) .* numel(holo);

    holo_rec{1} = RAAR_nf(sqrt(double(holo)), ones(p.rec_height), p.num_iterations,...
        p.F, p);
end

%% compare with frc
p.beta = 7;

% FSC is a general function to calculate the Correlation in 2d and 3d
frc_holo_to_ori = FSC(angle((sample)), angle((holo_rec{1})), p);


%% CDI data setup
id_FT = abs(DFT(sample)).^2;
id_FT = id_FT ./ sum(id_FT(:));
figure; imagesc(log10(abs(id_FT)));
%%
if(1)
  FT = imnoise((id_FT*p.num_photons.* numel(id_FT))*1e-12, 'poisson')*1e12;
  FT(FT == 0) = 2*eps;
  FT = FT ./ sum(FT(:)) .* numel(FT);
```

```
    [CDI_rec{1}] = RAAR_ff(sqrt(FT),...
        exp(1i .* rand(p.rec_height)), p.num_iterations, p);
end

%% compare with frc
p.beta = 7;

% in CDI we have to align the reconstruction with the original,
    since the reconstruction can be shifted in the reconstruction
    array
[err, aligned_sample] = dftregistration(fft2(angle(CDI_rec{1})),...
    fft2(angle(sample)), 5);
%              err
aligned_sample = (ifft2(aligned_sample));
frc_CDI_to_ori = FSC(angle((CDI_rec{1})), aligned_sample, p);

%% compare both
% make nice pictures of results
```
