# Supplementary figures and images for: The fluence–resolution relationship in holographic and coherent diffractive imaging
Source: J Appl Crystallogr. 2017 Mar 22;50(Pt 2):531–8. doi: 10.1107/S1600576717003065 (PMC5377347; doi:10.1107/S1600576717003065)

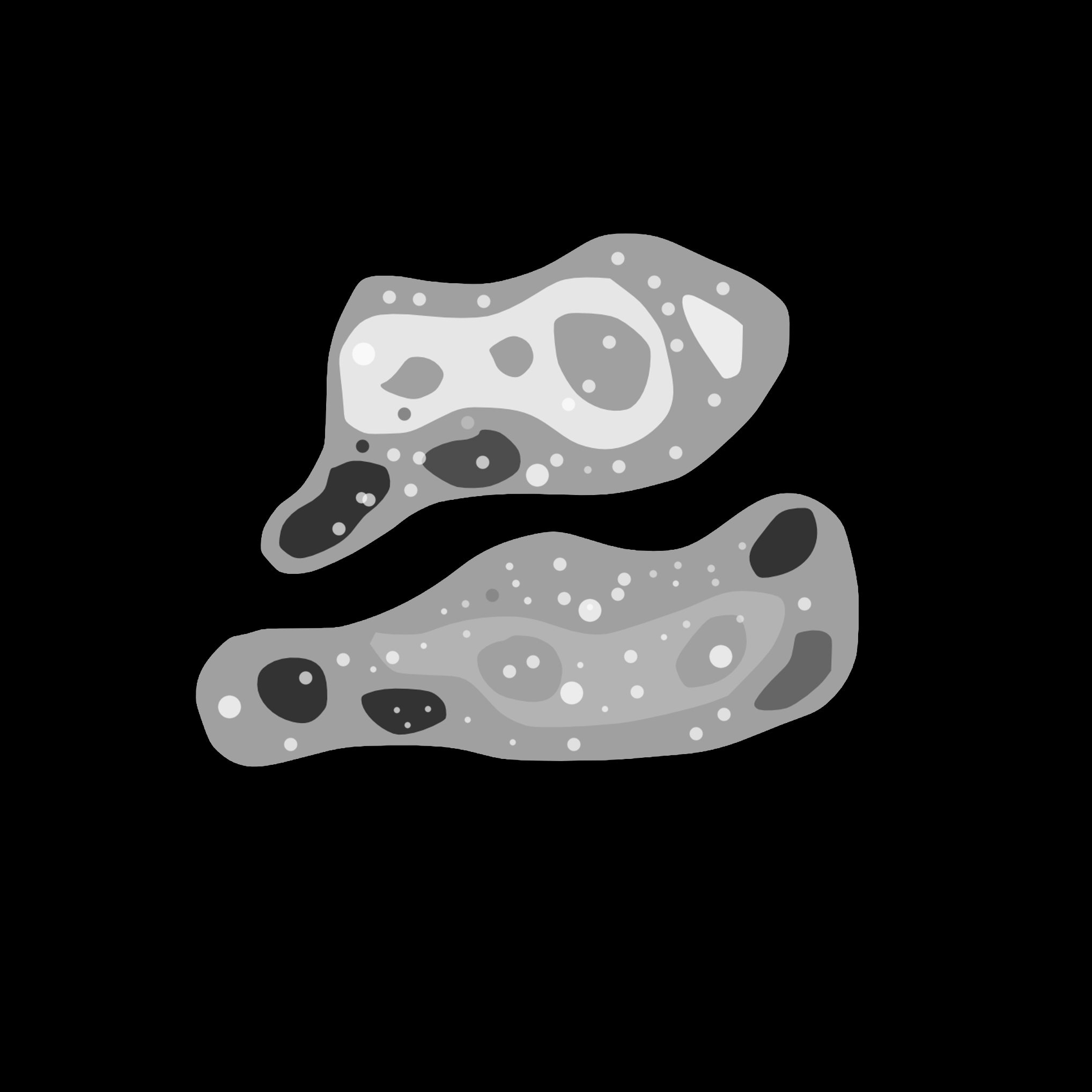

Supplement: Supplementary file 2 [file j-50-00531-sup2.zip › Resolution-and-Fluence-master/Scripts/dicty_sketch.png]

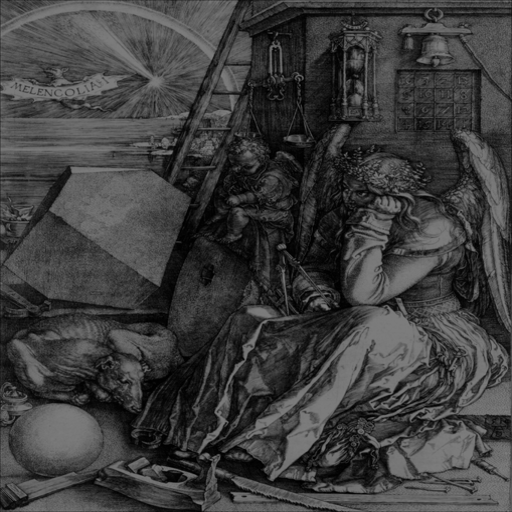

Supplement: Supplementary file 2 [file j-50-00531-sup2.zip › Resolution-and-Fluence-master/Scripts/durer.png]

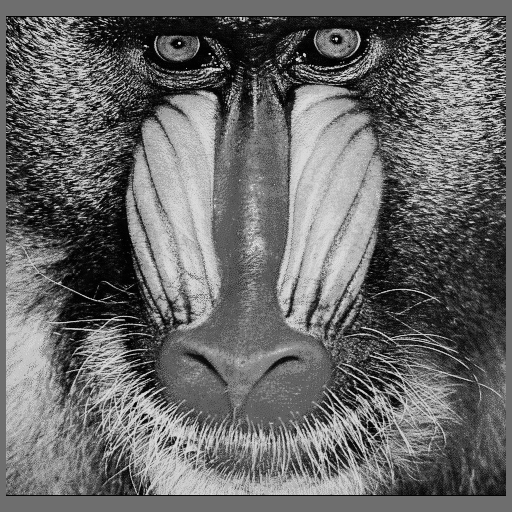

Supplement: Supplementary file 2 [file j-50-00531-sup2.zip › Resolution-and-Fluence-master/Scripts/mandrill.png]
